# Functional microbiome deficits associated with ageing: Chronological age threshold

**DOI:** 10.1111/acel.13063

**Published:** 2019-11-15

**Authors:** Susana Ruiz‐Ruiz, Sergio Sanchez‐Carrillo, Sergio Ciordia, María C. Mena, Celia Méndez‐García, David Rojo, Rafael Bargiela, Elisa Zubeldia‐Varela, Mónica Martínez‐Martínez, Coral Barbas, Manuel Ferrer, Andrés Moya

**Affiliations:** ^1^ Unidad Mixta de Investigación en Genómica y Salud Fundación para el Fomento de la Investigación Sanitaria y Biomédica de la Comunitat Valenciana (FISABIO) and Instituto de Biología Integrativa de Sistemas Universitat de València and Consejo Superior de Investigaciones Científicas (CSIC) València Spain; ^2^ CIBER en Epidemiología y Salud Pública (CIBERESP) Madrid Spain; ^3^ Instituto de Catálisis Consejo Superior de Investigaciones Científicas (CSIC) Madrid Spain; ^4^ Unidad de Proteómica Centro Nacional de Biotecnología Consejo Superior de Investigaciones Científicas (CSIC) Madrid Spain; ^5^ Centro de Metabolómica y Bioanálisis (CEMBIO) Facultad de Farmacia Universidad CEU San Pablo, Campus Montepríncipe Madrid Spain; ^6^ Departamento de Ciencias Médicas Básicas Facultad de Medicina Universidad CEU San Pablo Madrid Spain; ^7^ Present address: School of Natural Science Bangor University Bangor UK

**Keywords:** ageing, indole, metabolomics, microbiome, proteomics, tryptophan

## Abstract

Composition of the gut microbiota changes during ageing, but questions remain about whether age is also associated with deficits in microbiome function and whether these changes occur sharply or progressively. The ability to define these deficits in populations of different ages may help determine a chronological age threshold at which deficits occur and subsequently identify innovative dietary strategies for active and healthy ageing. Here, active gut microbiota and associated metabolic functions were evaluated using shotgun proteomics in three well‐defined age groups consisting of 30 healthy volunteers, namely, ten infants, ten adults and ten elderly individuals. Samples from each volunteer at intervals of up to 6 months (*n* = 83 samples) were used for validation. Ageing gradually increases the diversity of gut bacteria that actively synthesize proteins, that is by 1.4‐fold from infants to elderly individuals. An analysis of functional deficits consistently identifies a relationship between tryptophan and indole metabolism and ageing (*p* < 2.8e^−8^). Indeed, the synthesis of proteins involved in tryptophan and indole production and the faecal concentrations of these metabolites are directly correlated (*r* ^2^ > .987) and progressively decrease with age (*r* ^2^ > .948). An age threshold for a 50% decrease is observed ca. 11–31 years old, and a greater than 90% reduction is observed from the ages of 34–54 years. Based on recent investigations linking tryptophan with abundance of indole and other “healthy” longevity molecules and on the results from this small cohort study, dietary interventions aimed at manipulating tryptophan deficits since a relatively “young” age of 34 and, particularly, in the elderly are recommended.

## Graphical abstract

An “aged” microbiota is deficient in TrpB and TnaA proteins, which lead to a decrease in the production of tryptophan and the healthspan promoter indole. Dietary interventions aimed at manipulating the deficits of indole and tryptophan in the elderly is recommended.

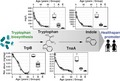

## 1 INTRODUCTION

The microbiota is now considered an additional organ in our body (Moya & Ferrer, 2016). Therefore, it undergoes changes throughout development, similar to other organs. Moreover, its physiological status, whether healthy or dysbiotic, influences the general health of individuals, although the directionality of this effect is not completely clear and is sometimes confusing (Kundu, Blacher, Elinav, & Pettersson, 2017). The numerous factors to which our body is exposed are also reflected by changes in the microbiota (Rojo et al., 2017). Various natural physiological changes are among the agents involved in modifying the microbial structure, both temporary (pregnancy or lactation) and permanent (the ageing process) (O'Toole & Jeffery, 2015). One of these changes is caused by chronological age. Humans are not free of microbes at birth, and the microbial community is continuously enriched and diversified with ageing (Odamaki et al., 2016). Indeed, it is generally accepted that a healthy and stable microbiota is established at the age of 3 years that remains similar until the adult stage, despite periodic fluctuations of various types (Odamaki et al., 2016). However, this conclusion should be re‐examined because the microbiota can be healthy throughout development but changes dynamically with age (Martí et al., 2017).

Based on 16S rDNA sequencing of the total gut microbiota, a relationship has been observed between clinical phenotypes in the elderly and an “aged microbiota” (Fransen et al., 2017). This microbiota is particularly enriched in pathobionts and displays a decreased abundance of bacteria with anti‐inflammatory and immunomodulatory properties (Fransen et al., 2017; García‐Peña, Álvarez‐Cisneros, Quiroz‐Baez, & Friedland, 2017; Kim & Jazwinski, 2018; Komanduri, Gondalia, Scholey, & Stough, 2019; Lu & Wang, 2018; Mangiola, Nicoletti, Gasbarrini, & Ponziani, 2018; Nagpal et al., 2018; Pasolli et al., 2019; Ramos‐Molina, Queipo‐Ortuño, Lambertos, Tinahones, & Peñafiel, 2019; Reveles, Patel, Forney, & Ross, 2019; Riaz Rajoka et al., 2018; Vaiserman, Koliada, & Marotta, 2017). These bacteria include the genera *Bacteroides*, *Alistipes*, *Parabacteroides*, *Faecalibacterium*, *Ruminococcus*, *Clostridium* clusters IV and XIVa, *Coprococcus*, *Roseburia*, *Coprobacillus*, *Anaerotruncus*, *Escherichia, Lactonifactor*, *Eubacterium*, *Lactobacillus*, *Bifidobacterium,* and *Akkermansia*, and families such as *Enterobacteriaceae, Eubacteriaceae*, *Porphyromonadaceae* and *Christensenellaceae*. However, most of these bacteria also represent microbial signatures of a number of diseases and disorders (Rojo et al., 2017). Therefore, researchers have yet to disentangle the dysbiotic alterations in those bacteria and their involvement in the ageing process. 

We would such as focus on the existing difference between the total and active microbiota (Moya & Ferrer, 2016). The total amount of bacteria at a given moment is different from the active working fraction, as this fraction has a functional role and is more relevant to the human health (Mills et al., 2019). In other words, although an examination of the temporal changes in the total (active and inactive) microbiota is interesting at any time scale, an evaluation of changes in the active members is more important in a broad sense (Moya & Ferrer, 2016) or in relation to ageing, life expectancy and age‐related diseases (Zierer, Menni, Kastenmüller, & Spector, 2015). As example, in their study of ageing and the microbiota, Sonowal et al. (2017) found that indoles produced from commensal active microbiota do not affect the fitness of young individuals but extend the healthspan of older individuals in diverse organisms such as *Caenorhabditis elegans*, *Drosophila melanogaster* and mice. During ageing, indoles induce the expression of host genes that promote healthy ageing. Thus, an assessment of microbial functions associated with active components of the human microbiota in well‐defined age groups is necessary. However, these investigations are still rare.

Our goal in the present manuscript was to identify the association between the functional gut microbiome and ageing and to identify potential functional deficits associated with ageing. Studies using proteomics and metabolomics provide direct valuable insights into these deficits compared to other “omics” techniques, but due to technicalities, studies in ageing research are limited to a few examples and small (*n* = 12) sample sizes (Gelfi et al., 2006; Zierer et al., 2015). In the present study, total proteins from bacterial cells isolated from the faecal material of three well‐defined age groups (*n* = 30) were subjected to shotgun proteomics; this approach allowed us to define the active fraction of the microbiota that synthesizes proteins. Subsequently, a functional analysis of the identified proteins was performed to assess presumptive age‐dependent functional deficits. Finally, using liquid chromatography coupled with mass spectrometry, functional deficits were experimentally validated in an extended set of replicate samples collected over time (*n* = 83). The combined analysis identified a reproducible microbiome biomarker associated with ageing, namely, a link between an elderly age and tryptophan and indole deficits. The relevance of tryptophan and indole to healthy ageing (Sonowal et al., 2017) and the results reported in this study will provide opportunities for the development of putative and innovative dietary strategies for healthy ageing.

## 2 RESULTS

### 2.1 General characteristics of the study population and study design

Faeces from ten infants (I), ten adults (A) and ten elderly individuals (E) were collected and analysed with a proteomic approach using a pooling strategy. Generally, sample pooling results in some unforeseen methodological and statistical bias. A recent proteomic study using individual and pooled serum samples from controls and patients with Creutzfeldt‐Jakob disease (CJD) revealed that compared to the analysis of individual samples, sample pooling affected the coefficients of variation of the minimum, maximum and mean values in both the control and CJD groups. However, the authors were able to identify biomarkers that significantly differed among groups (Molinari et al., 2018), which were then subjected to an in‐depth analysis using independent samples. This strategy was used in the present study because our main objective was to identify biomarkers of protein and functional deficits that substantially differed among the three well‐defined age groups (see Table 1). Briefly, pooling reduced the number of samples to analyse from 10 individual samples to two pools of five individuals for each of the three well‐defined age groups. Figure S1 summarizes the experimental groups analysed in this study. The idea was to establish a proteomic analysis with low resource and time requirements that would allow us to detect substantial differences among the three groups, which would be further validated using individual samples. (*NOTE: For the initial analysis, only time zero samples from each of the volunteers were measured, whereas for the validation analysis, samples collected at 0, 3 and 6 months were measured; see the *Section 4 *for a description of the sampling procedure*).

**Table 1:** Characteristics of the study population

Group	Age (median and IQR)	Weight (median and IQR)
Infant (I)	5 (2–5)	18 (15–20)
Adults (A)	34 (31–42)	69 (62–72)
Elderly (E)	67 (66–69)	67 (66–69)

Abbreviation: IQR, interquartile range.

### 2.2 An elderly status is associated with elevated levels of active bacteria

In total, 64,313 quality‐filtered nonredundant proteins were obtained from the faecal samples of all six pools, with a median value of 14,892 ± 1,875 proteins per pool (Table S1). Significant differences in the number of proteins were not observed among all six pools. This number is consistent with the average values reported in previous proteomic studies of the gut microbiota (Deusch et al., 2018; Mills et al., 2019; Serrano‐Villar et al., 2016). We calculated the alpha diversity parameters of active bacteria, namely, microbial richness (*d*), Pielou's evenness (*J*′) and Shannon index (*H*), from raw proteomic data using a previously described procedure (Deusch et al., 2018). As shown in Table 2, the richness of active species measured using d values was slightly increased with age, and the value of the E group was ca. 1.4‐fold higher than the I group. The number of species, as measured by calculating *J*′ and *H*, was also slightly increased with age. Thus, ageing is associated with a slightly greater protein diversity and richness of the corresponding active bacteria. Importantly, a statistical analysis was not performed because only two pools per group were analysed. However, the standard deviations among pools were very low and thus the differences observed were considered significant.

**Table 2:** Alpha diversity parameters of bacteria actively synthesizing proteins

Group	Diversity parameter of active microbiota^a^		
	*d*	*J*′	*H*′
I	934.7 ± 4.25	0.950 ± 0.009	7.95 ± 0.03
A	1,121.1 ± 26.2	0.951 ± 0.001	8.11 ± 0.26
E	1,330.5 ± 16.3	0.952 ± 0.001	8.29 ± 0.15

a Alpha diversity parameters were based on the analysis of proteins identified and quantified using MaxQuant data set for each of the six separate pools (two for I group, two for A group and two from E group). Results for each age‐group were given as mean values and the standard deviation of each of the two pools per age‐group.

### 2.3 Functional deficits associated with ageing

**Figure 1:**
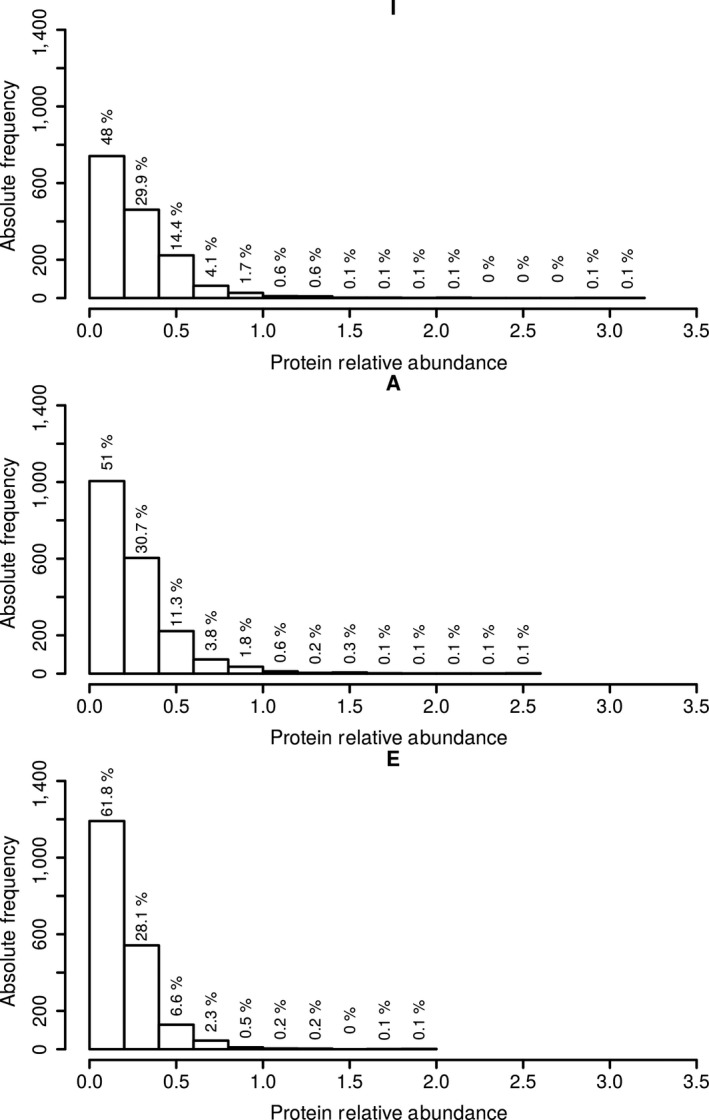
Distribution of relative protein abundances values in I, A and E groups. Represented is the number of proteins (*absolute frequency*) being expressed at different relative abundances (given as nemPAI or “ng protein per total µg of protein”) in the two pools per each of the three groups; the percentage of proteins per each expression group is given above the bars. Relative abundances represent the mean values of two pools of five individuals each for each of the three well‐defined age groups. The figure was obtained using R script

We performed the procedure described in the Section 4 to optimally evaluate the proteome of each of the two pools of the three well‐defined groups. In this experiment, only those proteins that were expressed in both pools from each of the three groups were considered to exhibit protein‐level changes due to ageing that were not due to inter‐individual variability. Three thousand four hundred seventy‐five of the 64,313 quality‐filtered nonredundant proteins met the following criterion: they were present in both pools, regardless of the relative abundance level. Those proteins were subsequently compared. Relative protein abundances were obtained using the procedure described in the Section 4. As shown in Figure 1, an exponential distribution of relative protein abundances was observed in all the three age groups, with a minor fraction of proteins (less than 1%) defined as super‐abundant. The abundance data thus suggest that the diversity of proteins being synthesized and expressed was not dominated by a particular type of protein or highly similar clusters of proteins, but consists of diverse proteins with similar abundances in the three well‐defined age groups investigated.

**Figure 2:**
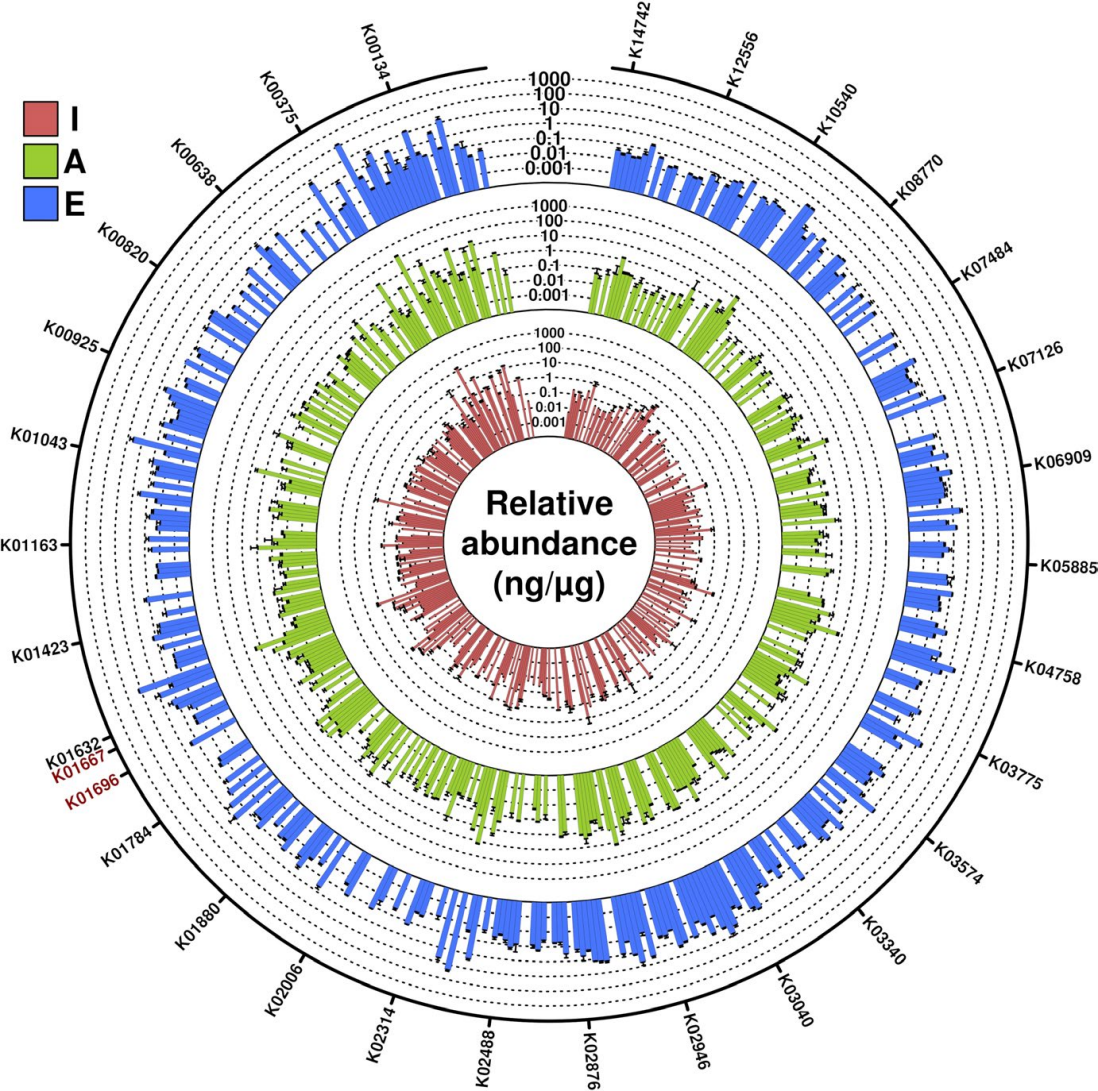
Distribution of the relative abundance of functional categories across I, A and E groups. Only the 3,475 quality‐filtered nonredundant proteins found to be expressed in the two pools of five individuals each for each of the three well‐defined age groups were accounted. The relative abundance of each KO was obtained by the sum of the relative abundances of all proteins assigned to each of the 437 identified KO. Inner circle represents data for I group, outer circle for E group and intermediate circle for A group. Relative abundances are colour‐coded as shown in the figure, with standard deviation (duplicates) shown. The KOs related to tryptophan and indole metabolisms are highlighted in red colour. The figure was created with the R language

**Figure 3:**
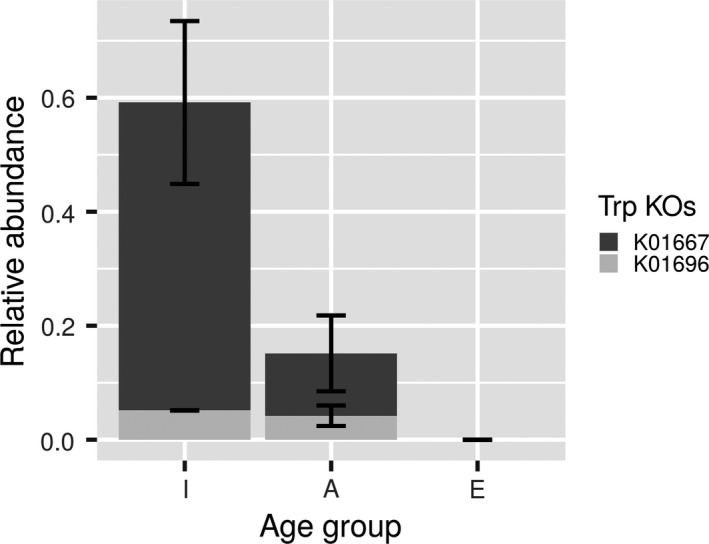
Distribution of total relative abundance of proteins (in ng/μg total protein) assigned to tryptophan metabolism in I, A and E groups. Only the nonredundant proteins assigned to tryptophan metabolism found to be expressed in the two pools of five individuals each for each of the three well‐defined age groups were considered. Data are the mean values of the two pools, with standard deviation shown. The figure was obtained using R script

**Figure 4:**
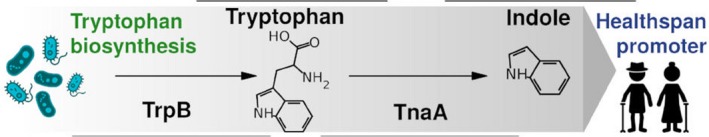
Microbiota‐based metabolism of tryptophan and indole. In brief, bacterial members of the microbiota synthesize tryptophan through TrpB tryptophan synthase; this metabolite is further degraded into the healthspan‐related indole by the action of TnaA tryptophanase

A subsequent examination of the 3,475 proteins revealed that only ca. 18% of the proteins were shared among the three well‐defined age groups (Figure S2). A sub‐set of ca. 14% was only expressed in infants, ca. 21% in adults and ca. 25% in the elderly, suggesting that ageing slightly and progressively induced the synthesis of proteins that are produced at levels below the detection limit in early life. Rather than examining the individual differentially expressed proteins among groups, which may be biased because of the biological variation after sample pooling, we performed a functional analysis based on the assumption of a representative functional deficit in the individual groups or samples; sample pooling has been shown suitable for these purposes (Molinari et al., 2018). The identified proteins were assigned to Kyoto Encyclopedia of Genes and Genomes (KEGG) Orthology (KO) pathways to assess the effects of ageing on different metabolic pathways defined by the KEGG. After calculating the relative abundance of each KO (see Section 4), no statistically significant differences were observed for most of the KO functions identified (Figure 2). Among the different KO annotations, we focused on those that discriminated among extreme age states, namely, pathways present in the I group and in the E group, or vice versa. By using this strict criterion, only the levels of proteins assigned to KO1667 (TnaA; tryptophanase) and KO1696 (TrpB; tryptophan synthase) were significantly increased in infants compared with adults and were below the detection limit in the elderly individuals (Figure 3). Most of these proteins belong to bacteria of the phylum *Firmicutes*. TrpB and TnaA are enzymes that catalyse the final steps in the biosynthesis of tryptophan and its further metabolism into indole (Figure 4). The observation that both proteins were undetectable in both pools of elderly individuals suggests that the capacity of an “aged” microbiota to produce both tryptophan and indole may be significantly reduced compared to the “infant” or “adult” microbiota.

### 2.4 Age‐dependent changes in tryptophan and indole metabolism

We validated the functional deficits detected using the pooling strategy by subsequently quantifying the relative abundance of TrpB (essential for the biosynthesis of tryptophan) and TnaA (converting tryptophan to indole) proteins in all individual I, A and E samples with targeted proteomics. We also quantitatively determined the tryptophan and indole concentrations in the faecal fluid from all volunteers. *(NOTE: This validation test was performed by using samples collected from most of each of the 30 individuals at 0, 3 and 6 months, for a total of 83 samples; please see the *Section 4 *for additional details)*.

**Figure 5:**
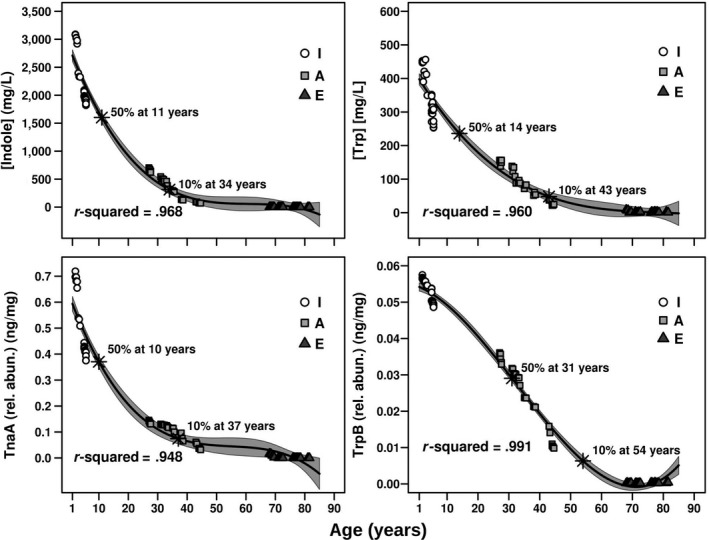
TnaA and TrpB relative abundances (in ng/μg total protein) and indole and tryptophan quantitative concentration (in mg/L faecal fluid) as a function of age. The data correspond to samples at time 0, 3 and 6 months of each of the 10 individuals and a total number of 83 samples—for details see Section 4. The fit was obtained using R script and the “Im” function, to extract a polynomial regression. Correlation and *r* ^2^ correspond to a polynomial regression model, whereas the grey zone represents the confidence value of 95% (meaning that the data within the grey are fit to the model with a confidence of 95%). Based on the fitting, the ages at which 50% and 90% reduction of initial values is reached are specifically shown

**Figure 6:**
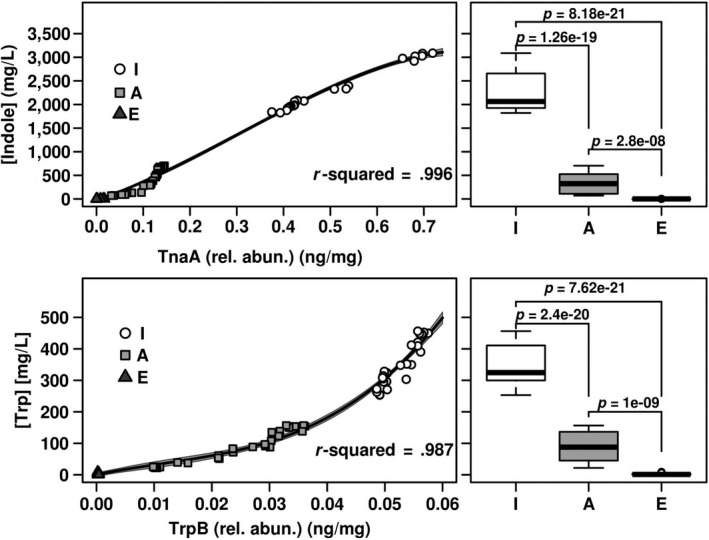
The relationship between TnaA and indole as well as TrpB and tryptophan abundances are responsible for the lower amount of both metabolites in elders. The samples considered and the data analysis (correlation and *r* ^2^) were as described in Figure 5. In the left panels, the fit for relationships between proteins and metabolites abundances is shown. In the right panels, box plot of the abundance of indole and tryptophan in the three well‐defined age groups of individuals examined (extracted from Figure 5) is shown. Statistical evaluation of the differences between groups was carried out by a Mann–Whitney *U* test

As shown in Figure 5, by plotting the relative TnaA and TrpB abundances as a function of age, we observed an exponentially decaying distribution (*r* ^2^ > .948). The concentrations of indole (ranging from ca. 3,088 to 0 mg/L) and tryptophan (ranging from ca. 456 to 0.44 mg/L) exhibited the same distribution with ageing (Figure 5; *r* ^2^ > .960), consistent with the relative abundance of the proteins implicated in their metabolism. Indeed, as shown in Figure 6 (left panel), correlations were observed between the abundance of TnaA and indole (*r* ^2^ = .996) and between TrpB and tryptophan (*r* ^2^ = .987). Based on the results from this analysis, TnaA, TrpB, tryptophan and indole were absent or detected at very low levels in elderly individuals, but were present at significantly higher concentration in infants and adults. The half‐life, defined as the amount of time required for half of the target proteins and metabolites to be degraded compared with their initial values, was calculated as ca. 11–31 years old (Figure 5). A greater than 90% reduction occurred from the ages of ca. 34–54 years. Consequently, statistically significant differences (*p* < 2.8e^−8^) in the faecal indole and tryptophan contents were observed between infant, adults and elderly individuals, which were significantly lower in the latter (Figure 6, right panel).

Undoubtedly, the elderly presents a microbiota that is unable to generate indole and tryptophan, or produces these metabolites at very low levels, and those signatures are among the best active biomarkers of chronological age identified to date.

## 3 DISCUSSION

In the present study, we comprehensively assessed the age‐associated changes in the gut microbiota, particularly the changes related to protein synthesis and eventually were able to distinguish among infants, adults and elderly individuals. Our study was performed using samples from three well‐defined age groups by analysing faecal shotgun proteomes and was further validated by classical analytical methods for metabolite quantification. Although ageing‐specific microbial species biomarkers have been identified (see Section 1), the identification of microbial deficits associated with ageing has not experienced the same increase in knowledge. This information is important to obtain an understanding of the roles of whole‐microbiome characteristics in ageing. An analysis of the proteome presents a distinct opportunity for studying the ageing‐associated microbiota by providing a functional perspective. Moreover, the identification of reproducible potential biomarkers of protein or functional deficits associated with ageing may facilitate the design of therapeutic or nutritional interventions.

Here, we have compared the proteins associated with the active fraction of the microbiota in infants, adults and elderly individuals. Ageing is associated with the progressive activation of gut bacteria, possibly because bacteria must react to increasing number of factors associated with preserving the health status in response to exposure to an increasing number of environmental conditions that are distinct and greater than the conditions experienced during early life stages. Most importantly, we identified a link between ageing and the microbial pathway associated with tryptophan and indole production and metabolism by the commensal microbiota. The key proteins involved in tryptophan‐to‐indole metabolism, TnaA and TrpB are both more abundant and expressed in the gut microbiota of infants. Both were expressed at significantly lower levels in adults and at even lower levels or below the detection limit in elderly individuals.

As shown in a recent study, indoles from commensal bacteria extend the healthspan of geriatric worms, flies and mice, and indoles may represent a class of therapeutics that improve the way we age, but not how long we live (Sonowal et al., 2017). The essential amino acid tryptophan, which is the least abundant in terms of its use in proteins, is provided by the diet or produced by gut bacteria, can cross the blood‐brain barrier under the influence of the gut microbiota (Sandgren & Brummer, 2018) and is a biosynthetic precursor for a large number of complex microbial natural products. Recent in vitro studies have indicated the decreased production, transport and catabolism of tryptophan in patients with a number of disorders, as its catabolism plays a crucial role in human T cell differentiation, the regulation of immune function, T_reg_ development and neurological function (Favre et al., 2010; Vujkovic‐Cvijin et al., 2013; Sandgren & Brummer, 2018). Therefore, the impaired development of the intestinal microbial ecosystem leading to a diminished capacity to produce indoles and tryptophan in elderly individuals because of the low expression of gut microbial proteins constituting the enzymatic machinery involved in their production may play a central role in determining the health status.

Actually, researchers have postulated that a chronological age threshold at which the composition of the microbiota suddenly changes does not exist, and changes occur gradually with time (O'Toole & Jeffery, 2015). This hypothesis is reinforced by the results from the present study, as we observed that the capacity to synthesize tryptophan and indole progressively decreased with the chronological age. However, compared with previous investigations, our study suggests a threshold or age at which the microbiota‐based metabolism of tryptophan and indole begin to be significantly reduced, which may have health‐related consequences on ageing if not treated accordingly. Indeed, based on our results, from the age of 11 years, the human gut microbiota may exhibit a decreased capacity to produce these metabolites, and from the age of 34 years, this capacity may be reduced by more than 90% compared to childhood. The results of this study reinforce the hypothesis that dietary supplementation with indole (Sonowal et al., 2017) and tryptophan exert a beneficial effect on elderly individuals because their gut bacteria exhibit an impaired capacity to produce these molecules required for extending the healthspan. This supplement can be administered beginning at the age of 11 years, at which time a 50% decrease in the production of these metabolites occurs, and particularly beginning at the age of 34 years, when a greater than 90% reduction occurs (Figure 5).

Notably, this study has two major limitations. The first one related to the pooling strategy applied, which as discussed also by Molinari et al. (2018), is a delicate issue. From a methodological point of view, it is worth mentioning the following advantages of the pooling strategy used herein to capture differences in the protein expression profiles between groups of people with different ages. The first one related to experiment time/cost as, based on our estimations, the pooling decreases the number of samples to be analysed and reduced cost and time by ca. 5‐fold. From a statistical point of view, pooling will only be valid if one focuses in proteins detected in all pools within a group. This so‐called core proteome representing each group (Zhang et al., 2014) will provide a statistical meaning for inter‐group comparisons and to select biomarkers which, in a second step, can be validated by target mass spectrometry in all individuals. Our results actually demonstrated this approach results in the detection of ageing biomarkers. Having said that, one of the main cons of the strategy, aside the lower statistical significance, if the results are not further validated in individual samples, is that one loses the intra‐group differences. The second one is the relatively low number of individuals investigated (30 in total). However, triplicate samples (with samples collected from each volunteer at up to 6‐month intervals) were analysed, which increased the statistical significance of the outcomes. An analysis of a larger number of participants and administering a nutritional intervention including indole and tryptophan supplements may help clarify the possible beneficial effects of these molecules on the healthspan in the elderly.

Our results actually demonstrated that ageing significantly decrease tryptophan content in our gut environment. It is noteworthy that a 90% reduction occurs at a still “young” age of 34 years. A justification of this threshold reduction remains unclear and needs further investigation. As well, age‐thresholds, if any, for other microbiota products need to be established and reasoned. Waiting for these issues to be further evaluated, a recent investigation demonstrated that some bacteria are important in predicting age, as the abundance and loss of certain bacteria associated with person's lifetime (Galkin et al., 2018). We thus think that the tryptophan deficiency from a certain age may be associated to the co‐occurring changes in microbial composition and ecological interactions within the gut. Correlation analyses from 16S rRNA and tryptophan composition in faeces will allow, in the future, detecting networks of co‐occurring bacteria and tryptophan level at different ages.

Whatever the case, tryptophan is known to play a fundamental role in health and neuroprotection (Fang, 2019; Platten, Nollen, Röhrig, Fallarino, & Opitz, 2019), as the kynurenine pathways use tryptophan to produce nicotinamide adenine dinucleotide (NAD^+^). This coenzyme is a longevity molecule, which can be metabolized by at least three types of proteins (PAPRs, sirtuins and CD38/CD157), with a side product of nicotinamide (NAM) (Blacher et al., 2019; Fang et al., 2017; Platten et al., 2019). The depletion of both NAD^+^ and NAM in human cells is detected in individuals with major neurodegenerative diseases, such as Alzheimer's and Parkinson's diseases, cardiovascular disease, muscle atrophy and cancer (Verdin 2015; Roager & Licht, 2018; Fang, 2019; Platten et al., 2019). From our results, it is thus plausible that the translocated amount of tryptophan from the gut environment to the human cells and their further capacity to produce NAD^+^ and NAM, may be reduced as we are getting older; this may influence a healthy longevity, if it is not counteracted. Although these levels remain undefined in tissue/cells of the individuals herein investigated, a recent investigation by Blacher et al. (2019) demonstrating that an impaired microbiota is a cause of reduced nicotinamide (NAM) in amyotrophic lateral sclerosis (ALS) patients and mouse models, agree with this scenario being plausible.

Based on the results presented and the importance of tryptophan and their side products, we suggest that nutritional intervention based on tryptophan supplementation may be required for a “healthy” longevity. The data suggest this intervention not being essential during infancy and early adulthood, as these essential molecules are produced by the corresponding gut microbiota at those ages, albeit at significantly lower level in adults. However, the “aged” microbiota in elderly individuals does not produce these molecules. A number of dietary factors and nutritional interventions with other products exert positive effects on the elderly (Ticinesi et al., 2017; Cortés‐Martín et al., 2018; Grosicki, Fielding, & Lustgarten, 2018; Sandgren & Brummer, 2018; Vemuri et al., 2018; Wu et al., 2018; Gao, Zhang, Huang, Shen, & Qin, 2018; Jones et al., 2019; Tran et al., 2019) or extend the lifespan of rodents (Miller et al., 2019). Thus, we are proposing a new intervention.

## 4 MATERIALS AND METHODS

### 4.1 Study participants and sample preparation strategy

Thirty healthy volunteers from Valencian Community (Spain) were involved in this study, and ten infants (I) were recruited between 2 and 5 years (age average 3.9 ± 1.45), ten adults (A) at 25–45 years (age average 35.4 ± 6.59) and ten elderly (E) at 65–85 years (age average 74.5 ± 4.25). Personal identification for clinical studies was encoded. All volunteers were asked to fill a questionnaire obtaining information about their diet, general health, medical history, vaccinations especially in the case of children and intake of antibiotics and other drugs. None had intestinal organic disorders, chronic diseases nor recent treatment with antibiotics. All procedures were reviewed and approved by the Ethics Committee of *Fundación para el Fomento de la Investigación Sanitaria y Biomédica de la Comunitat Valenciana* (FISABIO). Written informed consent was obtained from all subjects, or the parents in the case of children. For 24 volunteers, three faecal samples were collected following the instructions provided by the researchers (one sample at time 0, one sample 3 months later and one sample 6 months later). For five volunteers, two faecal samples were collected at time 0 and 3 months later, and for one volunteer, only the initial sample was collected. Together, a total of 83 samples were collected.

Faecal samples (10 g or ca. 10 ml) were collected in sterile containers containing 10 ml of RNAlater Solution (Ambion), to stabilize and preserve the integrity of RNA, until brought to the laboratory. Samples were homogenized adding 10 ml phosphate‐buffered saline (PBS) (containing, per litre, 8 g of NaCl, 0.2 g of KCl, 1.44 g of Na_2_HPO_4_ and 0.24 g of KH_2_PO_4_ [pH 7.2]) and then centrifuged to eliminate solid waste. *(NOTE: according to our protocol the faecal material was diluted three times: 10 g re‐suspended in 30 ml total volume)*. Samples, hereafter referred to as “*faecal suspension,*” were stored at −80°C in freezers until further processing.

For this study, three groups (I, A and E) were analysed after pooling. Five individual samples from the same group were pooled to constitute a pool (Figure S1). For that, 1‐ml of the “*faecal suspension*” of each of the five individuals were pooled to yield a total of 5‐ml. *(NOTE: only the initial samples (time 0) for each of the ten volunteers per groups were considered for pooling)*.

### 4.2 Protein extraction

The 5‐ml pool suspension obtained as above was lyophilized, and the freeze‐dried material was dissolved with chaotropic lysis buffer (7 M urea, 2 M tiourea, 5% 3‐[(3‐cholamidopropyl) dimethylammonio]‐1‐propanesulfonate and 10 mM 1,4‐dithiothreitol). In case of individual sample analysis (used for validation), 1‐ml of the corresponding individual “*faecal suspension*” was used. Then, 20 µg of protein was precipitated by methanol/chloroform method and re‐suspended in 20 µl of multichaotropic sample solution UTT buffer (7 M urea, 2 M thiourea, 100 mM triethylammonium bicarbonate (Sigma‐Aldrich)). The re‐suspended sample was reduced with 2 µl of 50 mM Tris(2‐carboxyethyl)phosphine hydrochloride, pH 8.0, at 37°C for 60 min, followed by addition of 1 µl of 200 mM cysteine‐blocking reagent methyl methanethiosulfonate (SCIEX) for 10 min at room temperature. Sample was diluted to 140 µl to reduce the urea concentration with 25 mM TEAB. Finally, digestion was initiated by adding 2 µg of Pierce MS‐grade trypsin (Thermo‐Fisher Scientific Inc.) to each fraction in a ratio 1:20 (w/w) and then incubated at 37°C overnight on a shaker. Sample digestion was evaporated to dryness in a vacuum concentrator.

### 4.3 Liquid chromatography and mass spectrometry analysis (LC‐MS)

Digested sample was cleaned‐up/desalted using Stage‐Tips with Empore 3M C18 disks (Sigma‐Aldrich). A 1 µg aliquot of resulting peptides was subjected to 1D‐nano LC ESI‐MS/MS (liquid chromatography–electrospray ionization tandem mass spectrometric) analysis using a nano‐liquid chromatography system Eksigent Technologies nanoLC Ultra 1D plus (SCIEX) coupled to high speed Triple TOF 5600 mass spectrometer (SCIEX) with a Nanospray III source. Analytical column used was a silica‐based reversed phase Acquity UPLC^®^ M‐Class Peptide BEH C18 Column (Waters Corporation). The trap column was a C18 Acclaim PepMap™ 100 (Thermo‐Fisher Scientific Inc.), 100 µm × 2 cm, 5 µm particle diameter, 100 Å pore size, switched on‐line with the analytical column. The loading pump delivered a solution of 0.1% formic acid in water at 2 µl/min. The nano‐pump provided a flow rate of 250 nl/min and was operated under gradient elution conditions. Peptides were separated using a 250 min gradient ranging from 2% to 90% mobile phase B (mobile phase A: 2% acetonitrile (Scharlab S.L), 0.1% formic acid (Sigma‐Aldrich); mobile phase B: 100% acetonitrile, 0.1% formic acid). Injection volume was 5 µl. Data were acquired using an ionspray voltage floating 2,300 V, curtain gas 35, interface heater temperature 150, ion source gas 125 and declustering potential 150 V. For IDA parameters, 0.25 s MS survey scan in the mass range of 350–1250 Da were followed by 35 MS/MS scans of 100 ms in the mass range of 100–1800. Switching criteria were set to ions greater than mass to charge ratio (*m*/*z*) 350 and smaller than *m*/*z* 1,250 with charge state of 2–5 and an abundance threshold >90 counts (cps). Former target ions were excluded for 15 s.

### 4.4 Proteomics data analysis and sequence search

Mass spectrometry data obtained were processed using PeakView v2.2 Software (SCIEX) and exported as mgf files which were searched using Mascot Server v2.5.1 (Matrix Science) against a high‐quality reference microbiome protein sequence database (Li et al., 2014; Pasolli et al., 2019; Zou et al., 2019). Sequences and ID codes, and functional and taxonomic assignations are detailed elsewhere (https://db.cngb.org/microbiome/genecatalog/genecatalog_human/; Li et al., 2014; Pasolli et al., 2019; Zou et al., 2019). Data acquisition of the total number of identified peptide spectra matched for a given protein (referred to as peptide‐to‐spectrum matching (PSM)), and Exponentially Modified Protein Abundance Index (emPAI), were calculated for each of the proteins. The PSM and emPAI can be used as a relative quantitation score of the proteins in a complex mixture based on protein coverage by the peptide matches in a database search result. Here, the emPAI was used (Arike & Peil, 2014). Normalized emPAI values (nemPAI) were obtained from the emPAI values by dividing each individual value by the sum of all emPAI values in a given experiment; then multiplied by 1,000 to calculate the relative protein abundance. *(NOTE: It is worth noticing that the total amount of protein used for proteomic analysis was set‐up to 1 μg, and therefore the nemPAI value can resemble a relative protein abundance in “ng protein per total μg of protein” (*Ferrer et al., 2018 *))*.

Diversity parameters of active bacteria, namely, those actively synthesizing proteins, were calculated from raw proteomic data as described previously (Deusch et al., 2018). Parameters were calculated for each of the six separate pools (two for I group, two for A group and two from E group), and the results for each age‐group were given as mean values and the standard deviation of each of the two pools.

When coarse‐grained data were required for protein and functional biomarkers discovery, the following procedure was applied: relative protein abundances were calculated separately for each of the six pools (two for I group, two for A group and two from E group), the two‐pools data were combined by only considering proteins that were present in both pools of the same age‐group, and the average protein abundance and the standard deviation were calculated. These filtered data sets were the ones used for coarse‐grained protein biomarker discovery analyses and for functional analysis of proteins, which was performed by assigning KO in order to analyse the varying abundance of specific proteins in certain pathways. KO assignations available elsewhere were used (Li et al., 2014; Pasolli et al., 2019; Zou et al., 2019). Relative abundance of each KO was obtained by the sum of the relative abundances of all proteins assigned to each KO (Ferrer et al., 2018).

In case of individual proteomic analysis, paired two‐sample *t* tests were used for pairwise comparisons of the relative abundance of proteins of interest (IBM SPSS Statistics, version 20.0. IBM Corp).

### 4.5 Determination of indole and tryptophan in faecal fluid

The following reagents and standards have been used: acetonitrile (LC‐MS grade, Sigma‐Aldrich) and DL‐tryptophan (Sigma‐Aldrich). All solutions were prepared using MilliQ^®^ water (Millipore).

A total of 1 ml of each of the “*faecal suspensions*” per individual samples were sonicated using a pin Sonicator^®^ 3000 (Misonix) for a total time of 20 s (10 W) on ice three times (with 20 s stop between cycles) and centrifuged at 15,000 *g* for 15 min at 4°C and the supernatant was retained and used for indole and tryptophan quantification. Prior to analysis, 50 µl of each supernatant were mixed with 200 µl of acetonitrile, vortexed (2 min) and centrifuged (10 min, 4°C, 15,000 *g*). The corresponding supernatants were transferred to analytical vials.

Indole was analysed using the Kovács reagent (Sigma‐Aldrich) as described elsewhere (Darkoh, Chappell, Gonzales, & Okhuysen, 2015)). For tryptophan determinations, a series of calibration samples were prepared from a solution of acetonitrile:water (4:1) spiked with different quantities of tryptophan. Each analysis was achieved using a liquid chromatography system consisting of a degasser, binary pump and autosampler (1260 Infinity II, Agilent Technologies) coupled to a triple quadrupole mass spectrometer (6470, Agilent Technologies). An Ascentis^®^ Express F5 column (50 × 2.1 mm, 2.7 µm, Sigma‐Aldrich) was maintained at 45°C during the analysis. The system was operated at a flow rate of 0.26 ml/min with solvent A (water) and solvent B (acetonitrile). The gradient was as follows: 0% B (0–1.4 min), 0%–100% B (1.4–1.8 min), 100% B (1.8–3.9 min) and 100%–0% B (3.9–4 min), keeping the re‐equilibration at 0% B for 6.5 min (11 min of the total analysis time). Data were collected in positive MRM mode. The MS‐ESI parameters were optimized as followed: capillary voltage was 4,000 V, gas temperature was 300°C, drying gas flow 10 L/min, nebulizer was 45 psi, sheath gas temperature 320°C, sheath gas flow 7 L/min and nozzle voltage was 2,000 V. MS‐QqQ parameters were as follows: fragmentor 80 V, dwell time 11 ms and cell accelerator voltage 7 V. The quantification transition was *m*/*z* 205–188, with corresponding qualification *m*/*z* 205–146. The analytical run was set‐up starting with the analysis of 5 injections of a pool of samples in order to equilibrate the chromatographic system, followed by injections of the calibration samples and then the samples in a randomized order. In between of them and after the final samples was injected, the series of calibration samples were injected again. Samples were maintained at 4°C throughout the analysis, and 0.1 µl of each was injected. Under our analytical conditions tryptophan elutes at 3.25 min. The corresponding peak areas were integrated by MassHunter Quantitative Analysis (B.08.00, Agilent). Their final concentration per sample was calculated based on the peak area for the corresponding standard in a calibration curve. The linearity of the relative response versus concentration was previously assessed under the same analytical conditions (*r* = .996, 0–10 mg/L; *r* = .995, 2.5–40 mg/L; *r* = .9996, 30–100 mg/L).

## CONFLICT OF INTEREST

The authors declare no competing interests.

## AUTHORS' CONTRIBUTION

S.R.‐R., M.F. and A.M. conceived and designed the experiments. S.R.‐R., and A.M. coordinated the samples collection. S.R.‐R., C.M‐G., E.Z‐V., S.C. and M.F. performed the proteomic experiments. D.R. and C.B. performed the analytics. S.R.‐R., S.S‐C., S.C., M.M‐M., R.B. and M.F. analysed the data. M.F. and A.M. wrote the paper. All authors reviewed and edited the manuscript. All authors have seen and approved the article; the corresponding author has full access to the data and had final responsibility for the decision to submit for publication. The authors wish to acknowledge the participation of all of the study participants who contributed to this work as well as the clinical research staff of the participating institutions who made this research possible.

## Data Availability

All data are available from the authors upon reasonable request.
