# Supplementary material for: Functional microbiome deficits associated with ageing: Chronological age threshold
Source: Aging Cell. 2019 Nov 15;19(1):e13063. doi: 10.1111/acel.13063 (PMC6974723; doi:10.1111/acel.13063)
Supplement: Supplementary file 1 [file ACEL-19-e13063-s001.docx]

**Ruiz-Ruiz et al. Supporting Information**

**FIGURE S1 Experimental groups used in this study.** To reduce the samples to be analyzed we generate 2 pools of 5 samples (time 0) per well-defined age group, as indicated. Following the identification of possible proteomic-based functional signatures using a pooling strategy, we analyzed also the individual samples extended to replicate samples taken 3- and 6-month after the initial sampling, so that together all constitute the validation group. Anonymized ID-numbers of each individual are indicated, with samples taken at time 0, and 3 and 6 months later being represented by “_1”, “_4”, “_7”, respectively. Asterisk indicates samples taken after 5 months of the initial sampling. Age of the volunteers at the initial sampling point is indicated in brackets. Abbreviations: I, infant; A, adult; E, elder; M, male; F, female.

**
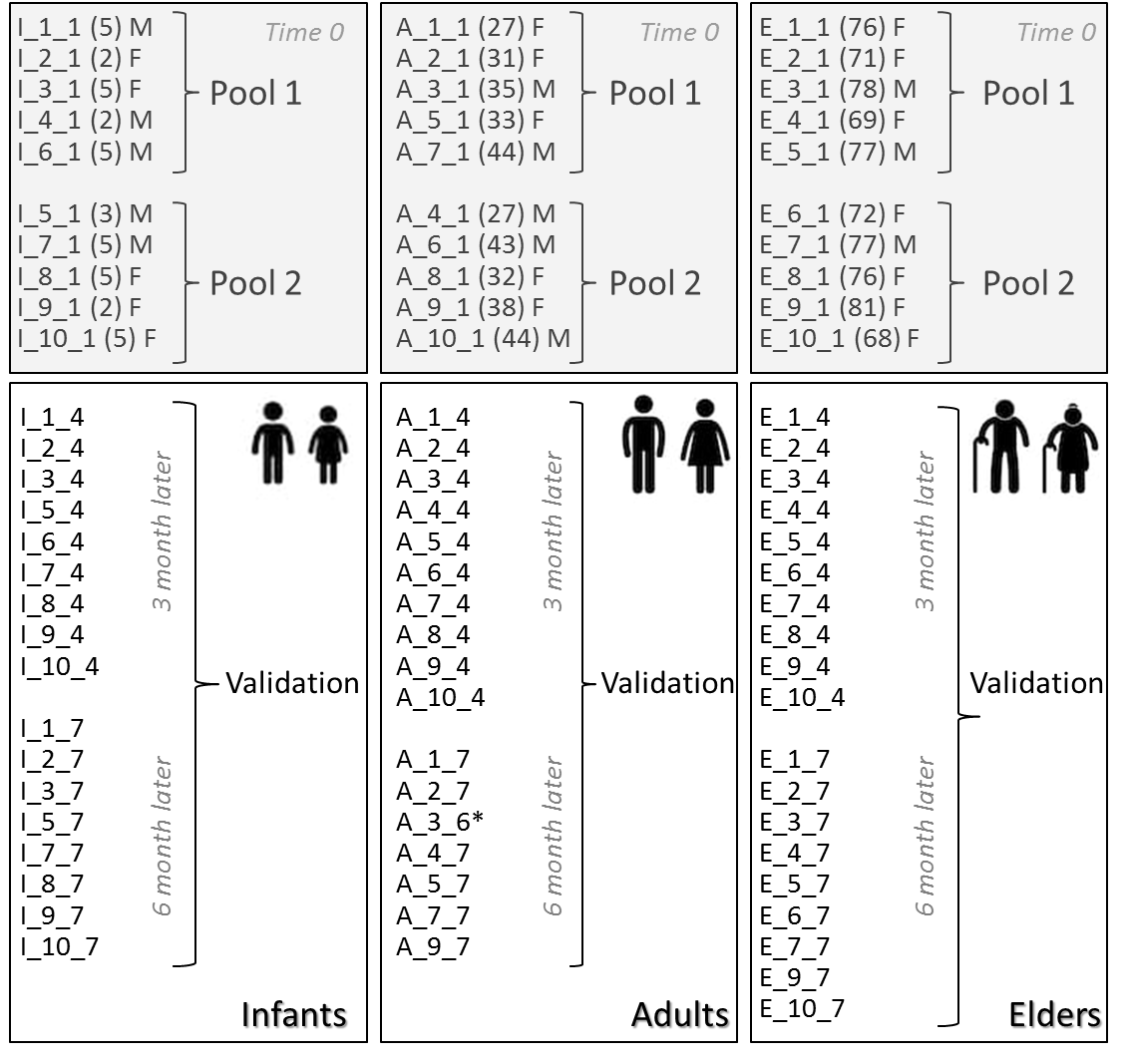
**

**FIGURE S2** **Venn diagrams indicating the percentage of proteins being shared among I, A and E groups.** Data according to the 3475 out of 64313 quality-filtered non-redundant proteins that passed the following criteria: they are present in the two pools (time 0 samples of 5 volunteer each) for each of the three well-defined age groups (I, A or E), whatever their relative abundance levels. The figure was obtained using R script.


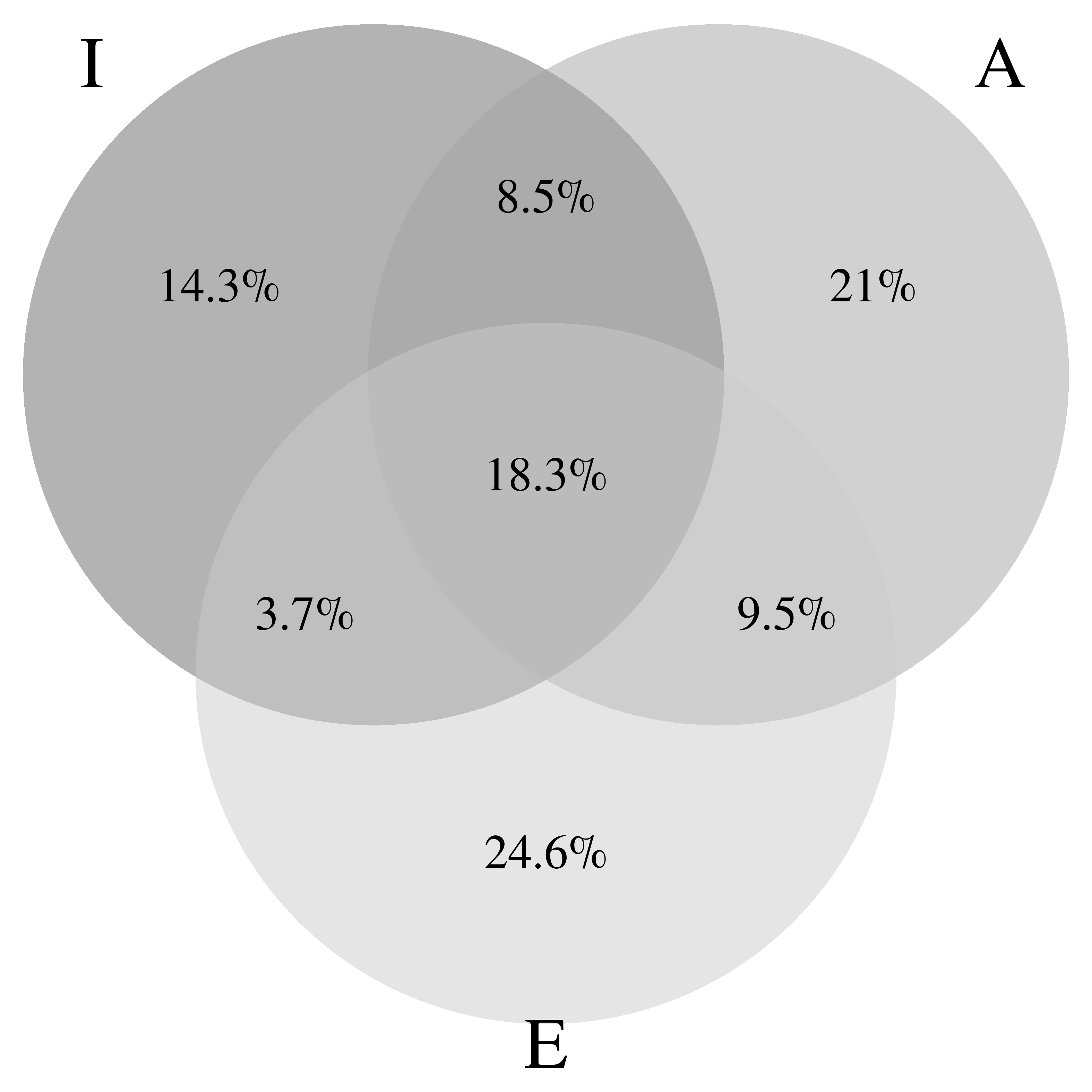


**TABLE S1** Quality-filtered non-redundant proteins identified from the faecal samples of all 6 pools from I, A and E groups (2 pools per each group), their expression level and their functional annotation. Proteomic information for: A) pool 1 of I group; B) pool 2 of I group; C) pool 1 of A group; D) pool 2 of A group; E) pool 1 of E group; F) pool 2 of E group; in all cases, Protein ID, protein description, molecular mass, isoelectric point (pI), protein score, number of peptide identified, PSM, emPAI and coverage are specifically indicated per each of the proteins. G) Proteomic information and annotation for 3750 quality-filtered non-redundant proteins meting the following criterion: they were present in both pools, regardless of the relative abundance level; information about gene length, gene status, cohort origin, taxonomic affiliation (phylum and/or genus level), frequency and individual frequency, KO, eggNOG, KO category and KO title are indicated. H) KEGG-KO functional assignations; relative abundance of each KO was obtained by the sum of the relative abundances of all proteins assigned to each KO. In all cases, functional annotations and protein information were as detailed elsewhere (<https://db.cngb.org/microbiome/genecatalog/genecatalog_human/>; Li et al., 2014; Pasolli et al., 2019; Zou et al., 2019). Because extensive size, this table is provided as separate Excel file.
