# Supplementary material for: Functional microbiome deficits associated with ageing: Chronological age threshold
Source: Aging Cell. 2019 Nov 15;19(1):e13063. doi: 10.1111/acel.13063 (PMC6974723; doi:10.1111/acel.13063)
Supplement: Supplementary file 3 [file ACEL-19-e13063-s003.docx]

**TABLE S1** Quality-filtered nonredundant proteins identified from the faecal samples of all 6 pools from I, A and E groups (2 pools per each group), their expression level and their functional annotation.
